# Post-Treatment Nutrition and Rehabilitation Support Is Associated with Reduced Psychological Distress After Early-Stage Upper Gastrointestinal Cancer Surgery: A Retrospective Cohort Study

**DOI:** 10.3390/healthcare14182936

**Published:** 2026-09-10

**Authors:** Mehmet Kadir Bartın, Müge Kara

**Affiliations:** 1 Department of General Surgery, Van Training and Research Hospital, University of Health Sciences, Van 65300, Türkiye; 2 Department of Internal Medicine, Van Training and Research Hospital, University of Health Sciences, Van 65300, Türkiye

**Keywords:** esophagectomy, gastrectomy, sarcopenia, propensity-score matching, survivorship care, dumping syndrome, Hospital Anxiety and Depression Scale

## Abstract

**Background/Objectives:** Curative-intent surgery for early-stage upper gastrointestinal (GI) cancer causes substantial nutritional, functional, and psychological morbidity; whether an integrated post-treatment nutrition and rehabilitation pathway improves distress and survival is unclear. **Methods:** We conducted a retrospective cohort study of 130 patients undergoing curative-intent esophagectomy or gastrectomy for early-stage esophagogastric cancer: 65 received a structured post-treatment nutrition and rehabilitation support program (Group A), and 65 received standard care (Group B). The primary outcome was psychological distress trajectory over 12 months (Distress Thermometer; secondarily HADS); overall survival was secondary. Propensity-score matching assessed covariate balance; a multivariable Cox model estimated the adjusted mortality association. **Results:** Distress scores were similar at 1 month (7.2 vs. 7.4; *p* = 0.43) and diverged progressively (group × time interaction *p* < 0.001; 4.9 vs. 6.1 at 12 months; *p* < 0.001, Cohen’s d ≈ 0.8; 95% CI for the 12-month between-group difference 0.65–1.75 points); HADS-A and HADS-D followed a concordant trajectory (8.4 vs. 10.1; 7.7 vs. 9.3; both *p* ≤ 0.003). Baseline covariates were reasonably balanced before matching (all standardized mean differences [SMDs] < 0.16) and met the prespecified <0.10 balance threshold for most covariates after matching. Fourteen deaths occurred (3 vs. 11); log-rank testing showed a significant survival difference (*p* = 0.021), consistent after matching (*p* = 0.037), and in the adjusted Cox model, support-program receipt was associated with lower mortality (hazard ratio 0.25, 95% CI 0.07–0.90; *p* = 0.034), though underpowered (14 vs. ~37 events required) and hypothesis-generating. **Conclusions:** Structured post-treatment nutrition and rehabilitation support was associated with markedly lower psychological distress, the prespecified primary outcome, with the achieved sample exceeding the a priori requirement for the assumed effect size; a secondary, exploratory survival association requires prospective confirmation. These findings support prospective, multicenter evaluation of integrated psycho-oncology and nutrition-rehabilitation care pathways after upper GI cancer surgery and should not be interpreted as demonstrating a causal treatment effect.

## 1. Introduction

Esophageal and gastric cancer remain among the most burdensome GI malignancies to treat curatively: even after early-stage, margin-negative resection, esophagectomy and gastrectomy leave a recovery period as much about relearning how to eat, move, and cope as about oncologic surveillance, with common sequelae including rapid weight loss, sarcopenia, dumping syndrome, micronutrient deficiency, and clinically significant distress. Despite guideline-level consensus that perioperative nutrition, structured exercise, and psychosocial screening each improve recovery [1,2,3], most postoperative pathways deliver these components in isolation rather than as a coordinated program—the gap motivating this study. This study’s primary hypothesis concerned psychological distress; overall survival was assessed only as a secondary, exploratory endpoint (Section 2.5).

Psychological distress after esophagogastric cancer surgery is common, persists for at least two years, and is linked to poorer quality of life [4], with prevalence and severity varying by cancer type/site [5]; its trajectory may itself carry prognostic weight for survival [6]. Nutritional decline and sarcopenia are independently linked to worse oncologic outcomes [7]; dumping syndrome remains under-recognized without a stepwise protocol [8,9]; and structured exercise improves function and may mitigate treatment-related morbidity [1,10]—evidence largely studied separately, leaving open what happens when combined.

Our institution implemented a structured post-treatment nutrition and rehabilitation support program coordinating surgical oncology, dietetics, physiotherapy, speech-language pathology, oncology nursing, and psycho-oncology around a shared follow-up timeline through 12 months. Patients on this pathway received scheduled nutrition screening and early feeding, a stepwise dumping-syndrome protocol, micronutrient monitoring, a structured exercise prescription, and routine distress screening (Distress Thermometer and HADS at 1, 3, 6, and 12 months) with survivorship care planning; patients outside this pathway received standard follow-up, with supportive care obtained only through individual referral.

This study compared psychological distress and overall survival between patients receiving this structured program and standard care, using propensity-score methods to account for measured baseline differences. We hypothesized structured support would lower distress and improve survival; the distress-score hypothesis was prespecified as the primary comparison, and the achieved sample size was subsequently found to exceed the a priori requirement for the assumed medium effect size (Cohen’s d = 0.5); survival was treated as secondary and exploratory given the modest expected number of deaths over roughly two years.

## 2. Materials and Methods

### 2.1. Study Design and Setting

We performed a retrospective cohort study of patients undergoing curative-intent surgery for early-stage esophageal, gastroesophageal-junction (GEJ), or gastric adenocarcinoma. Patients were assigned to Group A (structured program) or Group B (standard care) based on the pathway actually received: Group A comprised patients referred to and enrolled in the program after its institutional rollout (implemented institution-wide on 3 March 2025), with access determined by program capacity and clinician referral rather than patient-initiated selection; Group B comprised contemporaneous patients treated outside this pathway. Data were extracted from a structured institutional follow-up dataset (surgery dates December 2024–14 July 2026 cutoff), following STROBE guidance [11] (Appendix A).

For all participants, follow-up time for the survival analysis was defined from the date of curative-intent surgery—not from referral to or enrollment in the structured program—and this common index date was applied identically to Group A and Group B. Because survival time was anchored to surgery rather than to program entry, patients were not required to survive to a later start point to be counted as exposed, which removes the specific form of immortal-time bias that would arise from starting follow-up at program entry rather than at surgery. This does not, however, eliminate immortal-time bias entirely: because only patients who survived long enough to be referred to and enrolled in the program could be classified as Group A, and enrollment occurred during the index hospital admission, at a median of 3 days (IQR 1–6 days) after surgery, patients who died in the interval between surgery and enrollment could not be classified as Group A even though their survival time—counted from surgery—falls within that interval. Given this short surgery-to-enrollment interval, the residual immortal-time-bias risk is small, but is not zero. We therefore moderate our original statement: anchoring the time origin to surgery avoids the bias that would result from a late start of follow-up at program entry, but unless exposure status is fixed at or immediately after surgery, it does not by itself fully eliminate immortal-time bias arising from the surgery-to-enrollment interval; the landmark analysis discussed below and in Section 4.3 is intended to address this residual concern directly. Group B comprised contemporaneous patients who underwent surgery within the same overall study period, so that secular changes in surgical and perioperative practice would be expected to affect both groups similarly. To evaluate temporal comparability more directly, we also compared the distribution of surgery dates between the two groups: median (IQR) surgery date was 15 October 2025 (12 June 2025–20 February 2026) in Group A vs. 8 June 2025 (22 January 2025–14 November 2025) in Group B, indicating a modest rightward shift in Group A consistent with its post-rollout definition, with substantial overlap between the two interquartile ranges. We discuss the resulting potential for residual calendar-time/secular-trend confounding in Section 4.3. The program’s institutional rollout date is reported in Section 2.1. We did not perform a landmark or time-varying-exposure analysis; because Group A patients necessarily survived to program enrollment, a landmark analysis excluding the early post-surgical interval would provide additional reassurance against immortal-time bias and is noted as a limitation (Section 4.3).

### 2.2. Participants

**Figure 1:**
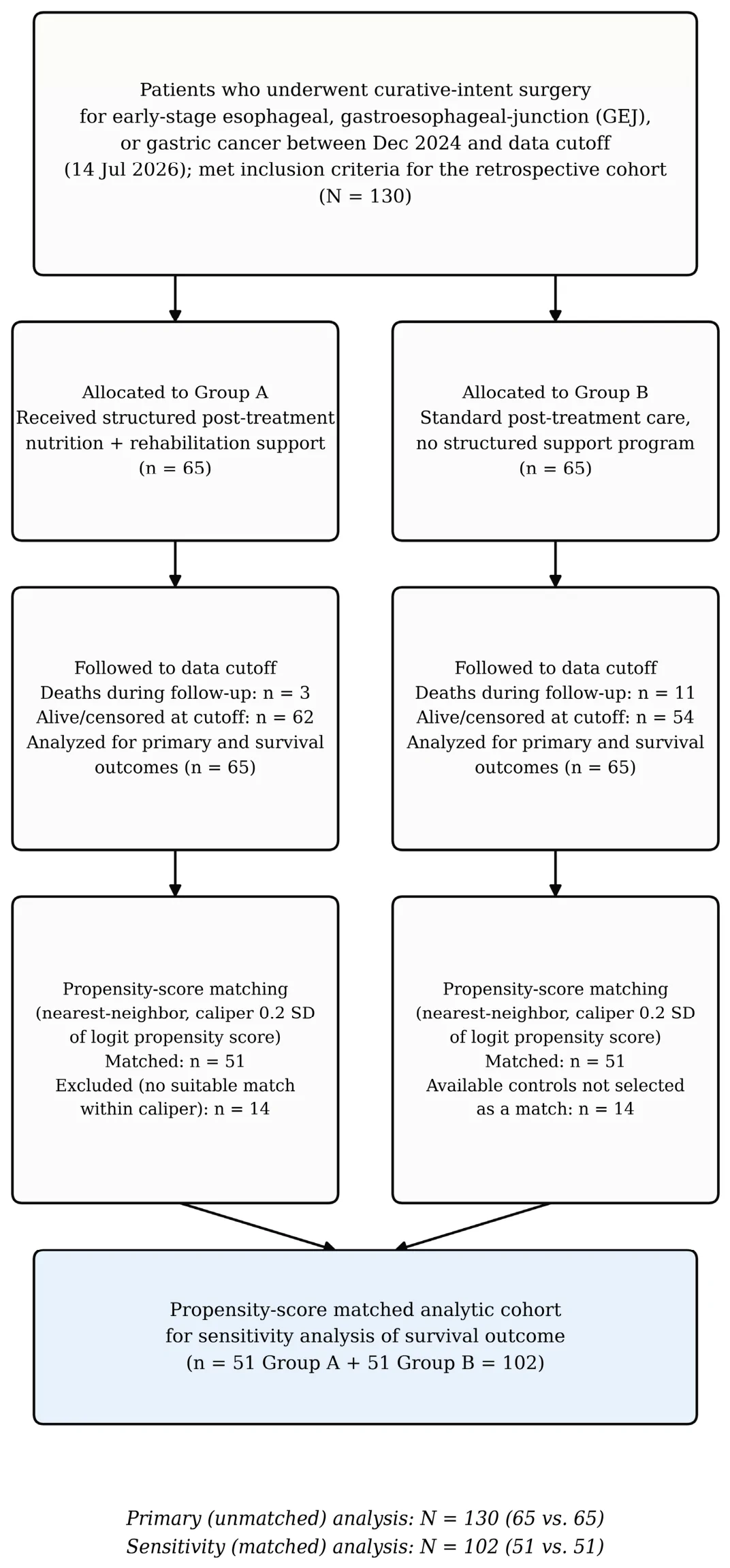
Patient selection flow diagram.

The analytic cohort comprised 130 patients (65 per group; Figure 1). Inclusion required curative-intent esophagectomy, total gastrectomy, or subtotal gastrectomy for early-stage disease, with or without neoadjuvant chemotherapy/chemoradiotherapy, and a recorded data-cutoff date. “Early-stage” was defined as clinical/pathological stage I–III (non-metastatic, resectable) disease amenable to curative-intent surgery; stage III was included because it is still treated with curative intent in this setting, whereas stage IV (metastatic) disease was excluded. Diagnosis deterministically defined surgical procedure (esophageal/GEJ → esophagectomy; gastric → gastrectomy), so surgery type was not entered as a separate covariate, avoiding collinearity.

This study was conducted per the Declaration of Helsinki and approved by the Ethics Committee of Health Sciences University Van Training and Research Hospital (protocol 2025/27-12; approved 25 June 2026); all patients had provided written general consent for use of de-identified data, and the Committee separately approved this retrospective analysis (see Institutional Review Board Statement).

### 2.3. Intervention

The structured support program followed a standardized, manualized protocol with a fixed visit schedule (full protocol in Supplementary Protocol S1): multidisciplinary involvement (surgical oncology, dietitian, physiotherapist, speech-language pathologist, nurse navigator, psycho-oncology/social work, primary care); nutrition screening (NRS-2002, MUST, or PG-SGA) [12] with early oral/enteral feeding and, when indicated, jejunostomy feeding, per ESPEN guidance [3]; structured dietary practice and a stepwise dumping-syndrome pathway (diet → viscosity modification/acarbose → octreotide → endoscopic/surgical revision) [9]; scheduled micronutrient monitoring (B12, ferritin/iron, folate, calcium, vitamin D); an aerobic/resistance exercise prescription (150 min/week aerobic; 2–3 days/week resistance), per exercise-oncology consensus [1]; and structured distress screening, survivorship care planning, and return-to-work support. Group B received standard postoperative follow-up outside the structured pathway, consisting of routine surgical outpatient visits per institutional protocol; dietitian, physiotherapy, and psycho-oncology input were available to Group B only through individual clinician referral rather than as a scheduled, protocolized component of care.

Distress Thermometer and HADS screening occurred at 2–4 weeks and 1, 3, 6, and 12 months. Patients screening positive (Distress Thermometer ≥ 4, or HADS-A/HADS-D ≥ 8; NCCN thresholds [2]) were referred to psycho-oncology within ~2 weeks, with monthly sessions for 3 months and thereafter as indicated (≤12 sessions/year); median sessions attended was 4 (IQR 3–6), addressing adjustment, coping skills, and behavioral activation, with referral to support groups or psychiatric care as indicated. The survivorship care plan, completed around 3 months by the nurse navigator with surgical/psycho-oncology teams, comprised a treatment summary, surveillance schedule, nutritional/exercise goals, return-to-work plan, and resource directory, provided to the patient and primary care.

### 2.4. Outcomes

The prespecified primary outcome was psychological distress trajectory on the 0–10 Distress Thermometer [13] at 1, 3, 6, and 12 months. As a secondary, convergent-validity measure, patients completed the Hospital Anxiety and Depression Scale (HADS) [14] (HADS-A, HADS-D; each 0–21, ≥8 = probable caseness). Secondary outcomes included overall survival, psycho-oncology referral, support-group participation, survivorship care plan completion, return-to-work status, self-reported health status, and nutritional/functional measures (weight loss, grip strength, gait speed, protein intake, exercise adherence), assessed at the 12-month follow-up visit. The Distress Thermometer and HADS were self-administered by patients on standard forms at each scheduled visit; treating clinicians were not blinded to group assignment, and formal independent (blinded) outcome assessment was not performed.

### 2.5. Sample Size and Power

A post hoc power calculation assessed whether the achieved sample could detect a medium effect (Cohen’s d = 0.5) for the distress-score difference; given the retrospectively assembled dataset, this is more accurately an achieved-power assessment than an a priori determination. Two-sided alpha 0.05 and 80% power required 64 patients/arm; the achieved 65/arm exceeded this. For survival, the Schoenfeld formula indicated ~37 events would be needed for 80% power to detect HR = 0.4; with only 14 deaths, survival is exploratory. Because this achieved-power assessment was performed after outcome data were observed, it describes whether the achieved sample was consistent with the assumed effect size rather than validating the primary finding, and it should not be cited as evidence of adequate power for the primary outcome (Section 4.1). HADS was a secondary, non-independently powered measure reported descriptively; the power calculation applies to the 12-month comparison only.

### 2.6. Propensity-Score Matching

**Figure 2:**
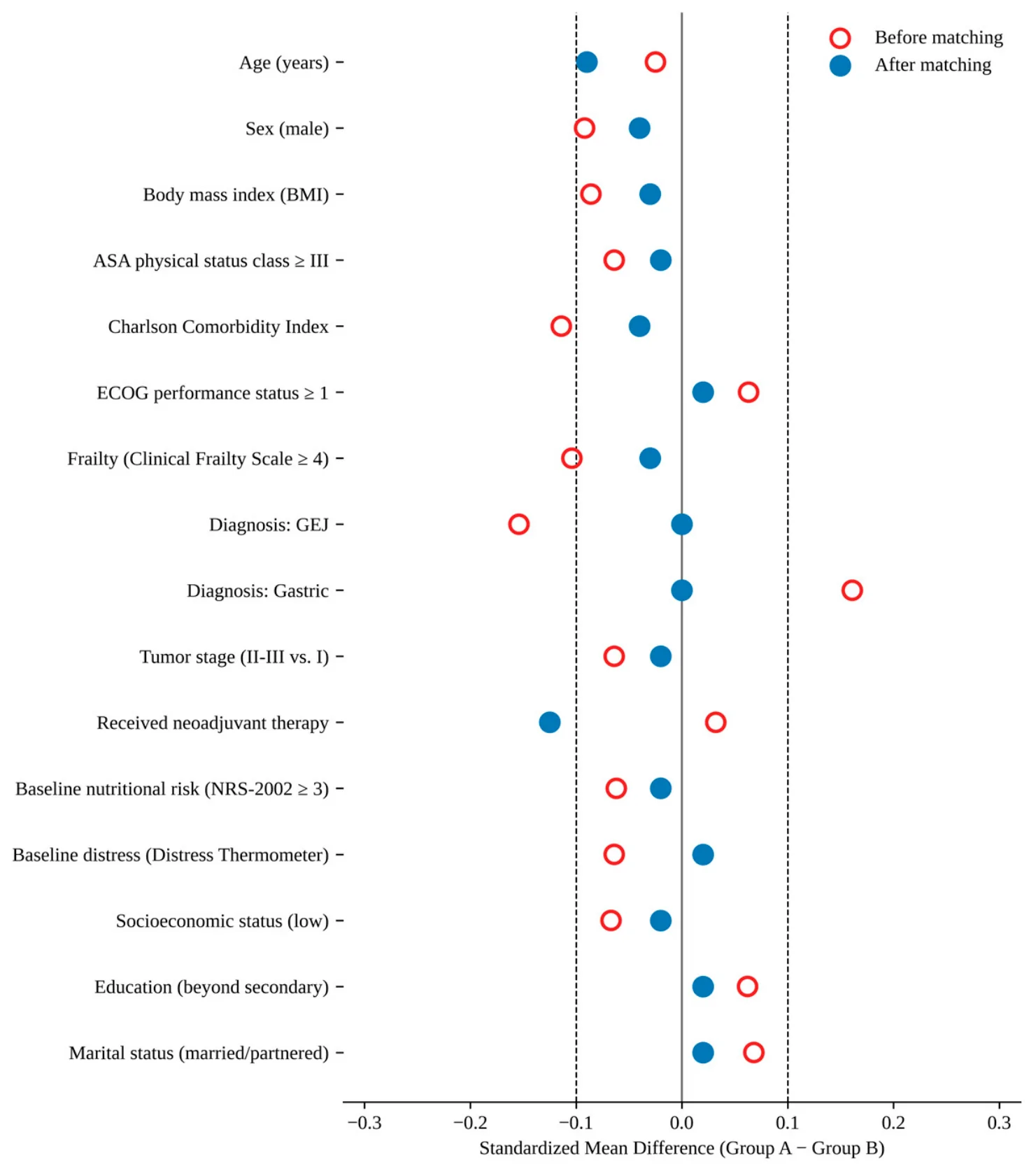
Love plot of standardized mean differences (SMD) before and after matching. Dashed lines mark the |SMD| = 0.10 balance threshold.

To assess and improve covariate balance, we estimated propensity scores for structured-program receipt using logistic regression on age, sex, BMI, ASA class, Charlson Comorbidity Index, ECOG status, frailty (Clinical Frailty Scale ≥ 4), diagnosis, tumor stage, neoadjuvant therapy, baseline nutritional risk (NRS-2002 ≥ 3), baseline distress, socioeconomic status, education, and marital status [15]. This set was used for the full-cohort (n = 130) model; continuous covariates (age, BMI, Charlson Comorbidity Index) were entered linearly, with no interaction or nonlinear terms. The Cox model for the rare survival outcome retained a parsimonious set (age, sex, diagnosis, neoadjuvant therapy) given only 14 deaths (~3.5 events/covariate, below the ≥10 guidance). Patients were matched 1:1 without replacement by greedy nearest-neighbor matching on the logit propensity score, caliper 0.2 SD. Balance was quantified by standardized mean differences (SMD < 0.10 = well balanced) and displayed graphically (Figure 2); per-covariate SMDs before matching are reported in Table 1.

### 2.7. Statistical Analysis

Continuous variables (mean ± standard deviation) were compared using *t*-tests (or Mann–Whitney U test when distribution assumptions were questioned); categorical variables (n, %) were compared using consistently applied chi-square tests. HADS-A, HADS-D, and HADS caseness were compared identically; Spearman correlations quantified Distress Thermometer–HADS concordance. Linear mixed-effects models (fixed effects for group, time, group × time interaction; random intercept per patient) tested whether distress trajectory differed by group for all three measures. The model specified a random intercept per patient with residual errors assumed independent and identically distributed conditional on the random intercept (i.e., a variance-component/compound-symmetry covariance structure), fit by restricted maximum likelihood (REML); formal diagnostic assessment of model assumptions (residual normality and homoscedasticity, e.g., via residual and Q–Q plots) was not performed, which we note as a limitation (Section 4.3). Overall survival was estimated by Kaplan–Meier (Greenwood CIs) and compared by log-rank test in both full and matched cohorts. A multivariable Cox model (adjusted for age, sex, diagnosis, and neoadjuvant therapy) estimated the hazard ratio for support-program receipt; adjusted HRs with 95% CIs are shown in a forest plot. Two-sided *p* < 0.05 was considered significant throughout, with no multiplicity adjustment, consistent with the exploratory status of all outcomes besides the primary endpoint. The Distress Thermometer trajectory was the sole confirmatory primary analysis; HADS and all remaining psychosocial, nutritional, functional, and survival comparisons are exploratory/hypothesis-generating and are labeled as such throughout. Analyses were performed using Python version 3.14.4, with pandas version 3.0.5 and NumPy version 2.4.6 for data handling, SciPy version 1.17.1 and statsmodels version 0.15.0 for statistical testing and mixed-effects models, lifelines version 0.30.3 for Kaplan–Meier, log-rank, and Cox regression analyses, and scikit-learn version 1.9.0 for the propensity-score logistic model. The proportional-hazards assumption of the Cox model was not formally evaluated (e.g., via Schoenfeld residuals) given the small number of events; this is noted as a limitation (Section 4.3).

### 2.8. Missing Data

For the longitudinal distress endpoints, no scheduled assessment was missing among patients alive and in follow-up; patients who died before a timepoint (cumulative deaths by 12 months: 2/65 Group A, 6/65 Group B, 8 total) contributed observations only up to their last assessment. Of the 130 patients, 122 (63/59) reached the 12-month primary follow-up and were analyzed at that timepoint; the remaining 8 had died before 12 months. The survival outcome had no missing values. Among these 122 patients, micronutrient panels had additional, visit-schedule-related missingness: 42 lacked a recorded 12-month panel, reflecting scheduling variation near the data cutoff, leaving 80 with a complete panel. These gaps did not affect the primary distress or survival analyses and are reported descriptively rather than imputed, per STROBE guidance (Appendix A, item 12c/14b). Deaths before the 12-month assessment (8 total: 2 Group A, 6 Group B) represent informative, non-random missingness: patients who died were, by definition, more clinically compromised, and their absence from later timepoints could bias the estimated between-group difference if mortality is related to distress trajectory. The mixed-effects model uses all available longitudinal observations under a missing-at-random assumption for dropout, which may not strictly hold when dropout is due to death; a formal joint (survival–longitudinal) or pattern-mixture sensitivity analysis for informative dropout was not performed, and this is noted as an explicit limitation (Section 4.3).

## 3. Results

### 3.1. Baseline Characteristics

Of 130 patients, 65 were included in each group according to the care pathway received (Figure 1; Table 1). Age, sex, BMI, ASA class, Charlson Comorbidity Index, ECOG status, frailty, diagnosis, tumor stage, neoadjuvant therapy, baseline nutritional risk, baseline distress, socioeconomic status, education, and marital status were all similar between groups (all *p* > 0.5; Table 1). All standardized mean differences were below 0.16 before matching and further reduced after matching for most covariates (Figure 2; Table 1); 51 matched pairs (102 patients, 78.5% of the cohort) were retained for the sensitivity analysis, with 14 Group A patients unmatched within the caliper. Per-covariate SMDs before matching are reported in Table 1; all were below the conventional 0.10 threshold except the gastric-diagnosis subgroup (SMD ≈ 0.16), consistent with the “all <0.16” summary above. Covariate-level *p*-values (Table 1) are reported for descriptive completeness only; consistent with current guidance for propensity-score studies, covariate balance was assessed using SMDs rather than the statistical significance of the treatment-assignment model (Table 1; Figure 2).

### 3.2. Primary Outcome: Psychological Distress

**Figure 3:**
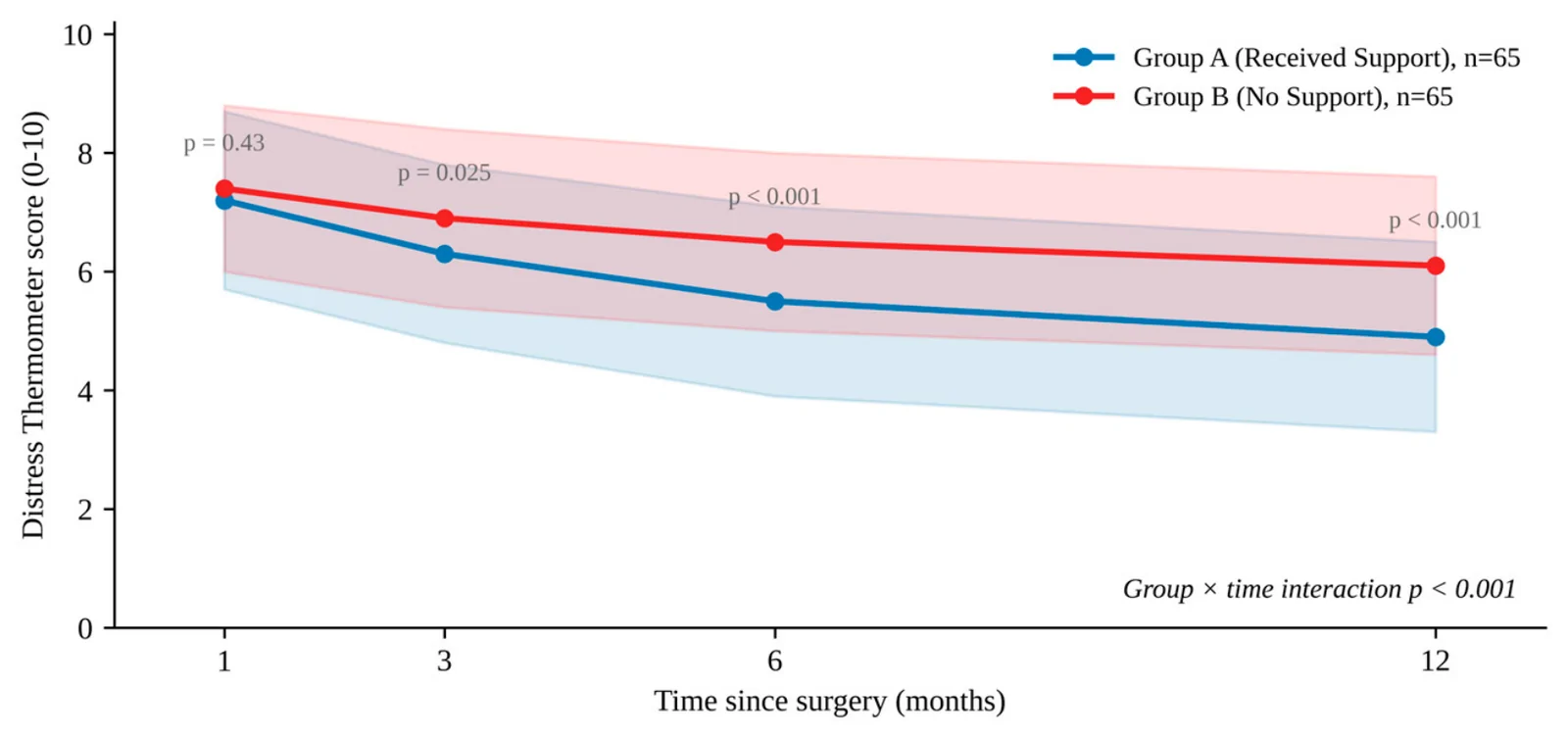
Trajectory of Distress Thermometer scores (mean ± SD) at 1, 3, 6, and 12 months, by group. The 12-month between-group difference corresponds to a 95% CI of 0.65–1.75 points. The shaded areas represent ±1 standard deviation.

Psychological distress followed different trajectories over 12 months (Table 2; Figure 3). At 1 month, before most program components were fully instituted, Distress Thermometer scores were similar (Group A 7.2 ± 1.5 vs. Group B 7.4 ± 1.4; *p* = 0.43). Group A scores then declined more steeply through 3, 6, and 12 months, widening from non-significant at 1 month to significant by 3 months (*p* = 0.025) and increasingly significant at 6 and 12 months (both *p* < 0.001; 12-month mean difference 1.2 points, Cohen’s d ≈ 0.8). A mixed-effects model confirmed a significant group × time interaction (*p* < 0.001), indicating the rate of improvement, not merely the endpoint value, differed between groups.

HADS followed a concordant trajectory, from near-identical 1-month values to significantly lower 12-month scores for HADS-A (8.4 ± 2.9 vs. 10.1 ± 3.2; *p* = 0.003; 95% CI for the between-group difference 0.61–2.79) and HADS-D (7.7 ± 2.6 vs. 9.3 ± 3.0; *p* = 0.002; 95% CI 0.60–2.60; interaction *p* < 0.001 for both), with less frequent 12-month caseness in Group A (55.6% vs. 74.6%; *p* = 0.028). The Distress Thermometer correlated moderately to strongly with HADS-A (ρ = 0.58) and HADS-D (ρ = 0.55; both *p* < 0.001), supporting convergent validity.

Group A was also more often referred to psycho-oncology/counseling, participated more in support groups, and completed a survivorship care plan more often (all *p* ≤ 0.047); median sessions attended was 4 (IQR 3–6). Self-reported health status was more often “Good” in Group A (*p* = 0.043); return-to-work completion was numerically higher but not significant (*p* = 0.096) (Table 3).

### 3.3. Nutritional and Functional Secondary Outcomes

Mean weight loss was lower in Group A (11.0% ± 5.8% vs. 14.5% ± 6.5%; *p* = 0.0015), with higher grip strength, gait speed, protein intake, and exercise adherence (all *p* ≤ 0.024) (Table 3), consistent with the distress-score results and suggesting the observed association was not confined to a single domain.

Distress Thermometer and HADS values at 12 months appear in Table 2; see Table 2 and Figure 3 for the complete trajectory.

### 3.4. Survival

**Figure 4:**
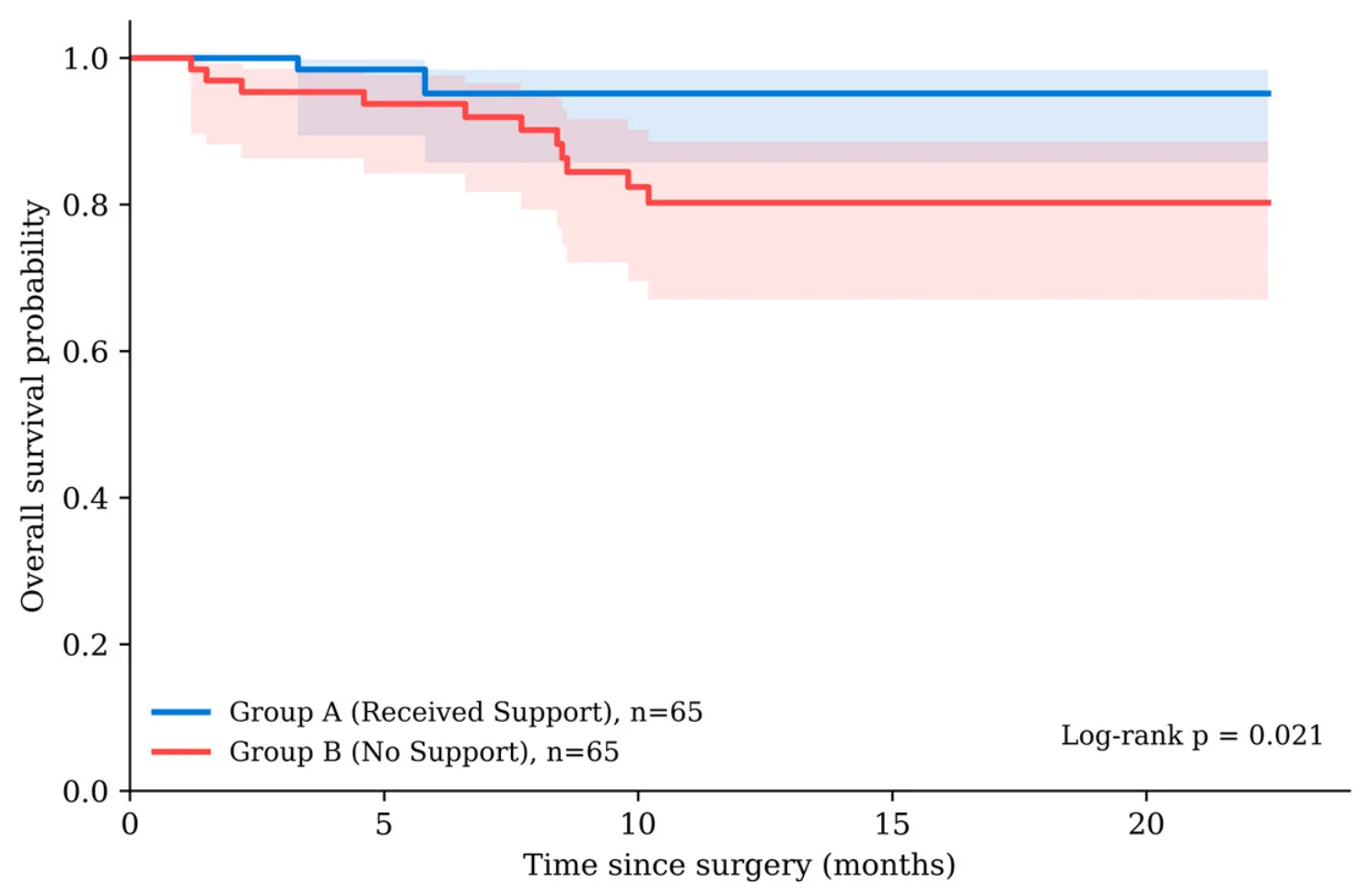
Kaplan–Meier estimates of overall survival by group, full (unmatched) cohort. The shaded areas represent the 95% confidence intervals.

**Figure 5:**
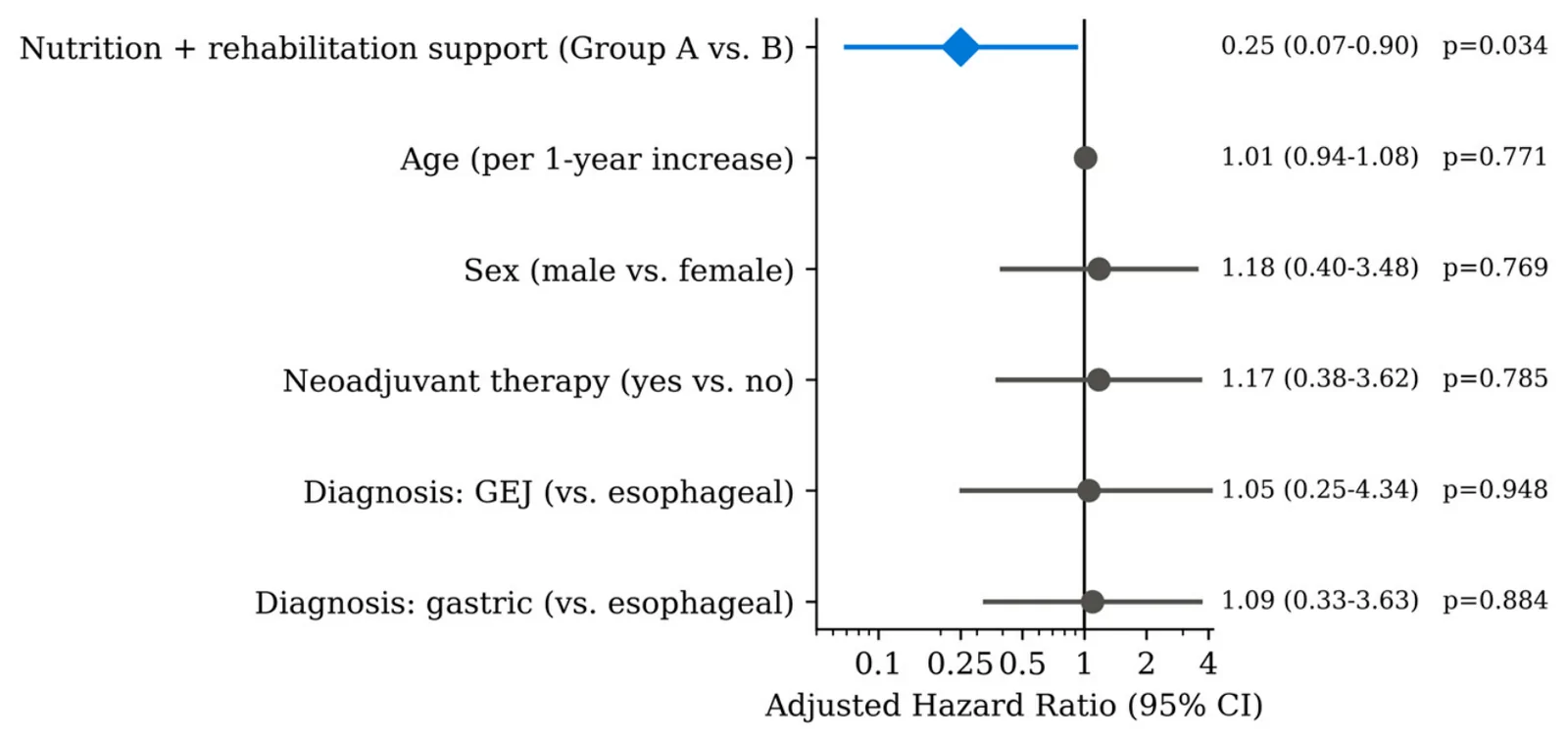
Forest plot of adjusted hazard ratios (95% CI) from the multivariable Cox proportional-hazards model. The blue rhombus represents the adjusted hazard ratio for the nutrition and rehabilitation support program, whereas the gray circles represent the adjusted hazard ratios for the other covariates; horizontal lines indicate 95% confidence intervals.

Fourteen deaths occurred: 3 in Group A and 11 in Group B. Kaplan–Meier-estimated survival diverged progressively after ~5 months (Figure 4); log-rank testing was significant in the full cohort (χ^2^ = 5.32, *p* = 0.021) and directionally consistent after matching (χ^2^ = 4.37, *p* = 0.037). In the adjusted Cox model (age, sex, diagnosis, and neoadjuvant therapy), support-program receipt was independently associated with lower mortality (HR 0.25, 95% CI 0.07–0.90; *p* = 0.034); none of age (HR 1.01, 0.94–1.08), sex (HR 1.18, 0.40–3.48), neoadjuvant therapy (HR 1.17, 0.38–3.62), or diagnosis (GEJ HR 1.05, 0.25–4.34; gastric HR 1.09, 0.33–3.63) reached significance (Figure 5). With only 14 events, well below the ~37 needed for 80% power to detect HR = 0.4, the wide CI reflects this limited event count. Given the small number of events relative to the four covariates in the adjusted model (~3.5 events/covariate), the point estimate (HR 0.25) is likely unstable and should not be interpreted as evidence of a treatment benefit; it is reported descriptively to motivate an adequately powered prospective study. Model stability under a more parsimonious specification or a penalized (e.g., Firth-type) Cox regression was not assessed and is noted as a limitation (Section 4.3). The finding should be interpreted as hypothesis-generating pending confirmation in an adequately powered, prospective, multicenter cohort.

### 3.5. Sensitivity and Internal Consistency

The direction and magnitude of the survival association were consistent in the full and matched cohorts (despite the latter losing 28 patients, 21.5%, to matching) and whether unadjusted or adjusted; no covariate other than group assignment approached significance. This offers only modest reassurance: because the unmatched, matched, and adjusted analyses draw on the same measured covariates within the same non-randomized cohort, their agreement does not constitute independent evidence against unmeasured confounding, and consistency across underpowered analyses is not equivalent to adequate power in any one of them.

## 4. Discussion

In this retrospective cohort of 130 patients undergoing curative-intent surgery for early-stage upper GI cancer, a structured, multidisciplinary post-treatment nutrition and rehabilitation support program was associated with lower psychological distress and better nutritional/functional recovery than standard care. A secondary, exploratory survival analysis also suggested a possible benefit but rested on only 14 deaths and should be regarded as hypothesis-generating. Baseline covariates were reasonably balanced before and after matching on measured variables (Table 1), which reduces, but does not eliminate, the possibility that these associations reflect differential case-mix rather than the structured program itself; propensity-score matching cannot account for unmeasured confounders such as clinician referral behavior, patient motivation, or social support (Section 4.3).

The 12-month distress-score difference (1.2 points; d ≈ 0.8) exceeded our power assumption (d = 0.5) and pooled effects for isolated psychosocial interventions [16], emerging gradually—indistinguishable at 1 month, diverging over the following 11 months (Figure 3; Table 2)—consistent with a cumulative, multicomponent effect rather than an immediate effect of enrollment. Group A also lost less weight and had better-preserved grip strength, gait speed, and protein intake, while concordant HADS-A/HADS-D reduction and a strong Distress Thermometer–HADS correlation support a genuine psychological effect. Because distress, nutrition, and function were measured concurrently and are mechanistically intertwined, our design cannot isolate which component contributed most; exceeding pooled psychosocial-intervention estimates [16] favors a cumulative contribution, consistent with protocolized screening and guaranteed referral outperforming unstructured help-seeking [17,18]. We therefore refer to the program as a multicomponent care pathway rather than as an intervention whose individual mechanistic contributions have been established.

Several hypothetical mechanisms may help explain the observed association; none were directly measured in this study, and they are proposed here as potential explanations rather than as demonstrated mediators. Plausible mechanisms for this gradual divergence include concrete milestones enhancing self-efficacy [19]; the exercise prescription as behavioral activation against postoperative withdrawal [20,21], consistent with higher adherence in Group A (59.0% vs. 47.0%; Table 3); psycho-oncology referral and survivorship planning supporting adaptive coping [22]; support-group participation increasing perceived social support [23,24,25]; and dumping-syndrome education reshaping illness perceptions [26]. None of these constructs was directly measured, so their relative contribution remains speculative, best disentangled by future factorial or SMART designs.

The survival finding warrants caution: the log-rank test was significant in both cohorts, and the adjusted HR (0.25) is consistent with benefit, but 14 deaths is well below what a prospective trial would require, and HRs from few events have wide CIs (0.07–0.90) and risk overestimation. We present survival as hypothesis-generating, not confirmatory; because the unmatched, matched, and adjusted analyses rely on the same observational cohort and the same measured covariates, their consistency is not independent evidence against confounding and is not a substitute for an adequately powered, prospective, multicenter study.

Our findings should be read alongside guideline recommendations already endorsing each component individually: nutritional optimization/early feeding [3,12], enhanced-recovery pathways [27], structured exercise [1,10], stepwise dumping-syndrome management [8,9], and distress screening [2]. What this study adds is an association between coordinated, protocolized delivery of these components and both a psychological outcome and survival, consistent with distress trajectory being prognostically relevant [6] and evidence of persistent post-surgical distress [4]. Sarcopenia is an established prognostic factor [7], and better-preserved grip strength, gait speed, and protein intake in Group A offer one plausible survival pathway alongside psychological/behavioral mechanisms.

### 4.1. Strengths

Methodological strengths partially offset the retrospective design: a single prespecified primary outcome assessed longitudinally, with the achieved sample size confirmed sufficient for the assumed effect size; propensity-score matching with a Love plot demonstrating balance; three consistent survival analyses; explicit quantification of the power shortfall; and STROBE-guided reporting (Appendix A).

### 4.2. Clinical and Research Implications

If confirmed prospectively, these findings would support embedding nutrition/rehabilitation support within standard postoperative pathways rather than individual referral. The program’s components are resource-modest and already guideline-recommended; coordinated delivery may exceed the sum of its parts. Future work should prioritize an adequately powered, multicenter trial with survival as a prespecified endpoint and factorial designs isolating each component’s contribution.

### 4.3. Limitations

This study has several limitations: a retrospective, single-center cohort with treatment-pathway assignment by program capacity and clinician referral rather than randomization, so unmeasured confounding cannot be excluded (potential unmeasured factors include clinician/surgeon preference, distance or access to care, patient motivation and adherence propensity, family/social support, baseline psychological resilience, postoperative complications, and the actual intensity of nutritional/rehabilitation participation received); an underpowered survival analysis with a wide CI and possible overestimation from few events; distress follow-up limited to four visits over 12 months; informative missingness from death before the 12-month assessment (survivor bias), which the mixed-effects model does not fully address (Section 2.8); single-center generalizability to other countries, smaller hospitals, resource-limited settings, and patients with more advanced disease, given that the multidisciplinary pathway depends on staffing and infrastructure that are not universally available; components delivered together, so no single driver can be isolated; per Supplementary Protocol S1 (Section S1.1), the implemented program omitted prehabilitation and dysphagia rehabilitation, and micronutrient monitoring ran only to 12 months—implementation, not protocol, limitations; the proportional-hazards assumption of the Cox model was not formally tested given the small number of events; nor were the linear mixed-effects model’s residual diagnostics (normality, homoscedasticity) formally examined (Section 2.7); a residual immortal-time interval between surgery and program enrollment (median 3 days) was not fully eliminated by anchoring survival time to surgery, and was only partially mitigated by the absence of a formal landmark analysis (Section 2.1); a modest calendar-time shift between groups (Group A surgery dates skewed somewhat later, consistent with sequential program rollout; Section 2.1), which could introduce secular-trend confounding not fully captured by measured covariates; and unblinded outcome assessors, raising possible detection bias; real-world implementation, staffing, and cost were not evaluated.

## 5. Conclusions

In this propensity-score-matched retrospective cohort, a structured post-treatment nutrition and rehabilitation support program was associated with lower psychological distress and better nutritional/functional recovery than standard care—the prespecified primary outcome, with an observed effect size (Cohen’s d ≈ 0.8) somewhat larger than assumed in the achieved-power assessment (d = 0.5). A secondary, exploratory survival analysis also suggested a possible mortality benefit, consistent across analyses but resting on few events and best treated as hypothesis-generating. These findings support prospective evaluation of coordinated nutrition and rehabilitation pathways within routine survivorship care and may inform the design of such pathways.

## Figures and Tables

**Table 1:** Baseline characteristics by group, before matching, including the standardized mean difference (SMD) for each covariate.

Characteristic	Group A (n = 65)	Group B (n = 65)	*p*-Value	SMD
Age, years, mean (SD)	63.6 (8.5)	63.8 (7.5)	0.887	0.025
Male sex, n (%)	32 (49.2%)	35 (53.8%)	0.599	0.092
Body mass index, kg/m^2^, mean (SD)	24.8 (3.6)	25.1 (3.4)	0.626	0.086
ASA class ≥ III, n (%)	22 (33.8%)	24 (36.9%)	0.714	0.064
Charlson Comorbidity Index, mean (SD)	3.4 (1.8)	3.6 (1.7)	0.516	0.114
ECOG performance status ≥ 1, n (%)	27 (41.5%)	25 (38.5%)	0.720	0.063
Frailty (Clinical Frailty Scale ≥ 4), n (%)	16 (24.6%)	19 (29.2%)	0.553	0.104
Diagnosis: Esophageal/GEJ/Gastric, n (%)	28 (43.1%)/11 (16.9%)/26 (40.0%)	29 (44.6%)/15 (23.1%)/21 (32.3%)	0.559	0.031/0.154/0.161
Tumor stage: I/II/III, n (%)	24 (36.9%)/29 (44.6%)/12 (18.5%)	22 (33.8%)/31 (47.7%)/12 (18.5%)	0.926	0.064/0.062/0.000
Received neoadjuvant therapy, n (%)	42 (64.6%)	41 (63.1%)	0.855	0.032
Baseline nutritional risk (NRS-2002 ≥ 3), n (%)	34 (52.3%)	36 (55.4%)	0.725	0.062
Baseline (preoperative) distress (0–10 DT), mean (SD)	6.9 (1.6)	7.0 (1.5)	0.714	0.064
Low socioeconomic status, n (%)	19 (29.2%)	21 (32.3%)	0.704	0.067
Education beyond secondary school, n (%)	28 (43.1%)	26 (40.0%)	0.722	0.062
Married or partnered, n (%)	47 (72.3%)	45 (69.2%)	0.700	0.068

**Table 2:** Trajectory of psychological distress (Distress Thermometer, HADS-A, HADS-D) at 1, 3, 6, and 12 months, by group. n analyzed (A/B): month 1, 65/65; month 3, 65/64; month 6, 64/62; month 12, 63/59 (reflecting deaths). Group × time interaction *p* < 0.001 for all three measures.

Measure, Timepoint	Group A	Group B	*p*-Value
Distress Thermometer (0–10)			
Month 1, mean (SD)	7.2 (1.5)	7.4 (1.4)	0.43
Month 3, mean (SD)	6.3 (1.5)	6.9 (1.5)	0.025
Month 6, mean (SD)	5.5 (1.6)	6.5 (1.5)	<0.001
Month 12, mean (SD)	4.9 (1.6)	6.1 (1.5)	<0.001
HADS-Anxiety (0–21)			
Month 1, mean (SD)	11.5 (3.0)	11.8 (2.9)	0.56
Month 3, mean (SD)	10.4 (3.0)	11.2 (3.0)	0.13
Month 6, mean (SD)	9.2 (3.0)	10.6 (3.1)	0.011
Month 12, mean (SD)	8.4 (2.9)	10.1 (3.2)	0.003
HADS-Depression (0–21)			
Month 1, mean (SD)	10.8 (2.8)	11.0 (2.7)	0.68
Month 3, mean (SD)	9.7 (2.7)	10.4 (2.8)	0.15
Month 6, mean (SD)	8.5 (2.7)	9.8 (2.9)	0.010
Month 12, mean (SD)	7.7 (2.6)	9.3 (3.0)	0.002

**Table 3:** Key psychosocial, nutritional, functional, and survival outcomes by group. Nutritional and functional measures (weight loss, grip strength, gait speed, protein intake, exercise adherence) were assessed at the 12-month follow-up visit.

Outcome	Group A (n = 65)	Group B (n = 65)	*p*-Value
HADS caseness at 12 months (either subscale ≥ 8), n (%)	35 (55.6%)	44 (74.6%)	0.028
Weight loss, %, mean (SD)	11.0 (5.8)	14.5 (6.5)	0.0015
Grip strength, kg, mean (SD)	27.0 (6.5)	24.5 (6.0)	0.024
Gait speed, m/s, mean (SD)	1.00 (0.23)	0.90 (0.22)	0.013
Protein intake, g/kg/day, mean (SD)	1.14 (0.28)	1.02 (0.26)	0.013
Exercise adherence, %, mean (SD)	59.0 (21.0)	47.0 (22.0)	0.0018
Referred to psycho-oncology/counseling, n (%)	34 (52.3%)	22 (33.8%)	0.034
Support-group participation, n (%)	30 (46.2%)	19 (29.2%)	0.047
Survivorship care plan completed, n (%)	33 (50.8%)	20 (30.8%)	0.020
Overall health status: Good, n (%)	28 (43.1%)	17 (26.2%)	0.043
Return-to-work: Completed, n (%)	19 (29.2%)	11 (16.9%)	0.096
Deaths during follow-up, n (%)	3 (4.6%)	11 (16.9%)	log-rank *p* = 0.021

## Data Availability

The data supporting this study’s findings are available on request from the corresponding author. The data are not publicly available due to privacy and ethical restrictions.

## Supplementary Materials

The following supporting information can be downloaded at: https://www.mdpi.com/article/10.3390/healthcare14182936/s1. Supplementary File: Supplementary Protocol S1, the multidisciplinary post-treatment nutrition and rehabilitation protocol underlying the structured support program (Group A), including Section S1.1 specifying which components were implemented/measured in the study versus provided as general background guidance. Refs. [1,2,7,9,10,12,27,28] are cited in the supplementary materials.

## Appendix A. STROBE Checklist for Cohort Studies

Completed according to the Strengthening the Reporting of Observational Studies in Epidemiology (STROBE) statement [11].

**Item**	**Section**	**Recommendation**	**Reported on**
1a	Title/Abstract	Study design named in title/abstract.	Abstract
1b	Title/Abstract	Balanced summary of methods/findings.	Abstract
2	Introduction	Background and rationale explained.	Introduction pp. 1–2
3	Introduction	Objectives/prespecified hypotheses stated.	Introduction p. 4
4	Methods	Key design elements presented early.	Section 2.1
5	Methods	Setting, locations, dates described.	Section 2.1 and Section 2.2
6a	Methods	Eligibility criteria, selection method given.	Section 2.2; Figure 1
6b	Methods	Matching criteria, exposed/unexposed n given.	Section 2.6; Figure 1 and Figure 2
7	Methods	Outcomes, exposures, confounders defined.	Section 2.3 and Section 2.4
8	Methods	Assessment source/method given per variable.	Section 2.4
9	Methods	Bias-mitigation efforts described.	Section 2.6; Discussion Section 4.3
10	Methods	Study-size rationale given.	Section 2.5
11	Methods	Handling of quantitative variables described.	Section 2.7
12a	Methods	Statistical methods, incl. confounding control.	Section 2.6 and Section 2.7
12b	Methods	Subgroup/interaction methods.	Not applicable—none prespecified
12c	Methods	Missing-data handling described.	Section 2.8
12d	Methods	Loss to follow-up addressed.	Section 2.8; Figure 1
12e	Methods	Sensitivity analyses described.	Section 2.6 and Section 2.7; Results Section 3.5
13a	Results	Numbers at each study stage.	Figure 1
13b	Results	Reasons for non-participation.	Figure 1
13c	Results	Flow diagram.	Figure 1
14a	Results	Characteristics and confounders reported.	Section 3.1; Table 1
14b	Results	Missing data per variable.	Section 2.8
14c	Results	Follow-up time summarized.	Section 2.5; Section 3.4
15	Results	Outcome events over time reported.	Section 3.4; Figure 4
16a	Results	Unadjusted/adjusted estimates with 95% CI.	Section 3.4; Figure 5; Table 3
16b	Results	Category boundaries for grouped variables.	Not applicable—continuous scale used
16c	Results	Absolute risk given alongside relative risk.	Section 3.4 (3/65 vs. 11/65)
17	Results	Other/sensitivity analyses reported.	Section 3.5
18	Discussion	Key results summarized vs. objectives.	Discussion p. 1
19	Discussion	Limitations, bias sources discussed.	Section 4.3
20	Discussion	Cautious, balanced interpretation given.	Discussion pp. 2–4; Section 4.2
21	Discussion	Generalizability discussed.	Section 4.3
22	Other information	Funding source stated.	Funding
