# Supplementary material for: Post-Treatment Nutrition and Rehabilitation Support Is Associated with Reduced Psychological Distress After Early-Stage Upper Gastrointestinal Cancer Surgery: A Retrospective Cohort Study
Source: Healthcare (Basel). 2026 Sep 10;14(18):2936. doi: 10.3390/healthcare14182936 (PMC13606524; doi:10.3390/healthcare14182936)
Supplement: Supplementary file 1 [file healthcare-14-02936-s001.zip › healthcare-4491430-supplementary.pdf]

Post-Treatment Nutrition and Rehabilitation in Patients with Early-Stage Upper Gastrointestinal Cancer

A practical, multidisciplinary protocol for the post-treatment care of patients with early-stage esophageal and gastric cancer following curative-intent surgery, with or without perioperative chemotherapy/chemoradiation.

This document is Supplementary Protocol S1 accompanying the manuscript "Post-treatment nutrition and rehabilitation support reduces psychological distress after early-stage upper GI cancer surgery" (Bartın MK, Kara M). It reproduces the multidisciplinary guidance underlying the structured support program (Group A) in Methods, Section 2.3, citing the general clinical literature on which Sections S2–S6 are based (see References). Section S1.1 clarifies which elements were implemented and measured in the study versus provided as general background guidance.

**Intended users**	Surgical oncology / GI surgery teams, medical oncology, dietitians/nutritionists, physiotherapists & exercise physiologists, speech-language pathologists, oncology nurses, primary care
**Patient population**	Adults with early-stage (resectable, non-metastatic) esophageal, gastroesophageal junction, or gastric cancer who have completed curative-intent treatment (surgery ± perioperative chemotherapy/chemoradiation)
**Scope**	Post-treatment (post-acute through long-term survivorship) nutrition management and physical/functional rehabilitation. Does not cover acute perioperative critical care or palliative/metastatic disease management
**Document date**	July 2026
**Associated manuscript**	Bartın MK, Kara M. "Post-treatment nutrition and rehabilitation support reduces psychological distress after early-stage upper GI cancer surgery" (see Supplementary Materials statement in the manuscript)

S1. Purpose and Scope

This protocol summarizes evidence-informed post-treatment nutrition and rehabilitation practice for early-stage esophageal, gastroesophageal junction (GEJ), and gastric cancer treated with esophagectomy or gastrectomy, generally combined with perioperative chemotherapy/chemoradiotherapy [1,8]. Surgery causes durable loss of gastric reservoir capacity, altered emptying, vagal denervation, and disrupted absorption, producing predictable nutritional and functional problems beyond discharge; a structured, multidisciplinary approach reduces malnutrition, readmission, muscle loss, and quality-of-life impairment, and supports tolerance of adjuvant therapy [1,3].

Sections S2–S3 cover team roles and nutrition; Section S4 covers rehabilitation and psychosocial support; Section S5 gives the follow-up timeline; Section S6 is a one-page quick-reference summary. This is an educational synthesis of published guidelines (see References), not a replacement for institutional protocols or clinical judgment.

S1.1 Relationship of This Protocol to the Associated Retrospective Cohort Study

The structured support program (Group A) in the associated cohort study implemented a subset of this guidance as a standardized program. Reconciliation with Sections S2–S6:

- Implemented and measured: nutrition screening/early feeding (3.1–3.3), dietary practice and the dumping-syndrome pathway (3.4–3.5), micronutrient monitoring (3.6), the exercise prescription (4.1), and distress screening with survivorship planning (4.4–4.5).
- Survivorship care plan: issued around 3 months in the study (Methods, Section 2.3), earlier than the 12-month timing shown in Section S5.

- Prehabilitation (Section S4.1): not implemented or evaluated — the study intervention was post-treatment only.
- Dysphagia rehabilitation (Section S4.3): SLP available for referral only, not a structured, measured program component.
- Micronutrient cadence (Section S3.6): measured only through 12 months in the study; the longer cadence shown reflects general practice.

S2. Multidisciplinary Team Roles

Core multidisciplinary roles [1,8]:

- Surgical oncology — complication assessment, feeding-tube oversight, stricture surveillance.
- Dietitian — screening, diet progression, enteral/parenteral planning, micronutrient monitoring.
- Physiotherapist/exercise physiologist — exercise prescription, sarcopenia management, functional testing.
- Speech–language pathologist — dysphagia assessment and rehabilitation.
- Oncology nurse navigator — care coordination, symptom triage, education.
- Psycho-oncology / social work — distress screening and support.
- Primary care — long-term surveillance once specialist follow-up decreases.

S3. Post-Treatment Nutrition

S3.1 Nutritional Assessment and Screening

Assess nutritional risk at diagnosis, before/after neoadjuvant therapy, at discharge, and each follow-up visit for ≥2 years [2,3].

- NRS-2002 [2], MUST, or PG-SGA at each visit; weight trend/% loss from baseline as bedside indicators.
- CT-based L3 muscle index where feasible — a strong predictor of complications and sarcopenia [3].
- Involve a dietitian proactively, not after weight loss is already established.

S3.2 Early Post-Treatment Feeding

Early oral/enteral feeding is supported after major GI surgery, individualized for upper GI anastomoses [1]:

- Begin within hours in stable patients; for esophagectomy (especially cervical anastomosis), individualize timing with the surgical team given a possible higher leak signal.
- Progress diet (liquids → soft → regular) by tolerance, not calendar; extra caution over age 75.
- Add parenteral nutrition if intake is <~50% of requirements for 3–4 days [1].

S3.3 Enteral (Jejunostomy) Feeding Management

A feeding jejunostomy (common at esophagectomy) bridges oral intake recovery.

- Supplement, not replace, oral intake once swallowing is safe; wean gradually.

- Monitor tube site, tolerance, and mechanical complications each visit.
- Remove once oral intake is stable at ≥75% of needs for 1–2 weeks with stable/improving weight.

S3.4 Practical Dietary Guidance After Esophagectomy/Gastrectomy

Practical guidance for a reduced/absent gastric reservoir, reinforced by a dietitian early [1]:

- 5–6 small, high-protein, energy-dense meals rather than 3 large ones.
- Eat slowly, chew thoroughly, stop at early fullness; avoid tough textures initially.
- Stay upright 45–60 min after eating; avoid lying flat 2–3 h; elevate head of bed at night.
- Separate fluids from solids by 30–60 minutes — important for dumping symptoms.
- Limit concentrated sweets, fatty/fried foods, carbonation, caffeine, and alcohol.
- Start a daily multivitamin at discharge; reassess micronutrients per Section S3.6.

S3.5 Managing Dumping Syndrome

Dumping syndrome results from rapid transit of hyperosmolar food into the small bowel after loss of pyloric/gastric reservoir function, a well-recognized complication of both esophagectomy and gastrectomy [4].

Early dumping (0–60 min after eating): fluid shifts into the bowel cause hypotension, tachycardia, sweating, and abdominal cramping/diarrhea.

Late dumping (1–3 h after eating): reactive hypoglycemia causes weakness, tremor, confusion, and diaphoresis.

Stepwise management [4]:

- First-line: small frequent meals, reduced simple sugars, adequate protein, separate liquids/solids by 30–60 min.
- Second-line: viscosity agents (guar gum, pectin, acarbose 50–100 mg with meals) for late dumping; GI side effects limit tolerance.
- Third-line: octreotide 25–50 mcg SC before meals after diet failure; long-acting formulations improve adherence. Monitor for steatorrhea/gallstones.
- Refractory: endoscopic or surgical revision with the surgical team.

S3.6 Micronutrient Monitoring and Supplementation

Micronutrient deficiency after esophagectomy or gastrectomy is common and often delayed, emerging months to years after surgery, so proactive scheduled monitoring is essential.

Nutrient	Why it's at risk	Typical supplementation	Monitoring
Vitamin B12	Loss of intrinsic factor / acid-producing gastric mucosa (especially total or near-total gastrectomy); reduced ileal exposure	350–1000 mcg oral daily, or 1000 mcg IM monthly if oral absorption unreliable	Serum B12 (± methylmalonic acid) every 6–12 months, lifelong after total gastrectomy
Iron	Duodenum/proximal jejunum bypassed; reduced gastric acid impairs non-heme iron absorption	≥45–60 mg elemental iron daily, dosed separately from calcium; IV iron if oral is ineffective/not tolerated	Ferritin/iron studies, CBC every 6–12 months
Folate	Reduced intake and altered absorption	400–800 mcg daily (often within a multivitamin)	Annually or with anemia work-up
Calcium	Duodenal bypass, vitamin D	1200–1500 mg/day in divided doses	Annually; with bone density

	deficiency, reduced dairy tolerance	(≤500 mg/dose); calcium citrate preferred	monitoring
Vitamin D	Fat malabsorption, reduced sun/dietary intake	≥800–3000 IU/day, titrated to serum 25(OH)D >30 ng/mL	25(OH)D annually
Fat-soluble vitamins A, E, K	Disrupted jejunal fat absorption after reconstruction	Individualized; often covered by a complete multivitamin	Assess if steatorrhea or malabsorption symptoms present
Zinc / copper	Roux-en-Y type reconstructions bypass main absorption sites	Individualized; avoid excess zinc without copper (can worsen copper deficiency)	Check if neurologic symptoms, poor wound healing, or refractory anemia

Suggested cadence: baseline at discharge, then 1, 3, 6, and 12 months, then every 6–12 months through at least year 5, extending indefinitely (lifelong for B12 and, after total gastrectomy, most listed nutrients). A complete multivitamin/mineral formulation (e.g., bariatric-strength) can reduce pill burden.

In the associated cohort study (see Section S1.1), micronutrient panels were measured and analyzed only through the 12-month primary follow-up window; the extended cadence above reflects general post-treatment practice beyond that study's analyzed window.

S4. Post-Treatment Physical and Functional Rehabilitation

S4.1 Structured Exercise Prescription

Cancer treatment (surgery plus perioperative chemotherapy/chemoradiation) causes a measurable, often prolonged decline in cardiorespiratory fitness and strength. Consensus exercise-oncology guidance recommends [5,6]:

Exercise type	Frequency	Intensity	Notes
Aerobic	3–5 sessions/week	Moderate intensity (~64–76% max HR or "somewhat hard" RPE); build toward 150 min/week	Walking, cycling, or swimming are well-tolerated starting points; start low and progress gradually post-treatment
Resistance	2–3 non-consecutive days/week	Moderate load, major muscle groups, 8–12 reps × 1–3 sets	Supervised or elastic-band home programs both show benefit; prioritize lower-limb and trunk musculature
Flexibility/functional	Daily as tolerated	Low intensity	Especially relevant after thoracotomy/thoracoscopy for range-of-motion and posture

Prehabilitation (exercise plus nutrition before/during neoadjuvant therapy) has trial evidence of benefit in esophagogastric cancer, better preserving functional walking capacity versus usual care [5,6]. Where feasible, begin at diagnosis, not only after surgery.

The study intervention associated with this protocol (see Section S1.1) was post-treatment only and did not implement or evaluate a prehabilitation phase.

S4.2 Sarcopenia Management

Sarcopenia (loss of muscle mass/strength) is common after upper GI cancer treatment and linked to worse outcomes [3].

- Identify via CT-based L3 measurement or grip strength/gait speed screening.
- Combined resistance-plus-aerobic programs over several months increase muscle mass versus non-exercising controls, best with high adherence (≥85%) [5,6].
- Combine with adequate protein (1.2–1.5 g/kg/day); exercise alone is less effective without nutritional support.

S4.3 Swallowing (Dysphagia) Rehabilitation

Dysphagia is common after esophagectomy due to altered anatomy, vagal injury, and anastomotic strictures.

- Refer to speech–language pathology when symptoms, aspiration risk, or slow oral progression appear.
- Exercise-based rehabilitation peri-/post-operatively is linked to earlier oral intake and shorter stay in available literature, though evidence remains limited — individualize per SLP assessment.
- Keep a low threshold for endoscopy to exclude anastomotic stricture, which is treatable and distinct from a functional swallowing problem.

S4.4 Psychosocial Support and Distress Screening

Upper GI cancer treatment carries a high symptom burden that elevates risk of distress, anxiety, and depression [7].

- Screen at diagnosis, major transitions, and follow-up visits; refer to psycho-oncology/counseling for significant scores [7].
- Address return-to-work planning, social eating anxiety, and caregiver burden proactively.
- Peer/survivorship groups can help normalize eating and lifestyle adjustments.

S4.5 Survivorship Care Coordination

As specialist visits decrease, formally transition surveillance:

- Provide a written survivorship/treatment summary and care plan (history, nutritional needs, surveillance schedule) to patient and primary care [8].
- Maintain oncology follow-up per surveillance protocols while shifting general health maintenance to primary care.

S5. Consolidated Follow-Up Timeline

Timepoint	Nutrition	Rehabilitation / Function	Psychosocial
Discharge	Dietitian-led diet plan; start multivitamin; jejunostomy teaching if applicable	Gentle mobilization; home exercise handout	Distress screen; caregiver education
2–4 weeks	Reassess oral intake/weight; wean jejunostomy feeds as tolerated	Begin structured walking; assess for dysphagia symptoms	Check adjustment, address early concerns
6–12 weeks	Weight/intake check; consider first micronutrient panel	Progress aerobic + start/advance resistance training; SLP follow-up if indicated	Reassess distress; return-to-work discussion
3–6 months	Micronutrient panel (B12, iron, folate, vitamin D, calcium)	Reassess functional capacity; sarcopenia screen	Ongoing screening
12 months	Full micronutrient panel; bone density if indicated	Reassess exercise program; consider formal fitness testing	Survivorship care plan issued*
Annually to 5 years, then as indicated	Micronutrient panel; ongoing dietitian access as needed	Encourage sustained exercise habits	Periodic distress screening

**In the study intervention, the survivorship care plan was completed earlier than shown, typically around the 3-month visit; see Section S1.1.*

S6. Quick-Reference Summary

Key practice points

- Screen nutritional status at every visit; involve a dietitian early, not after weight loss is already established.
- Individualize timing of oral/enteral feeding after esophagectomy with the surgical team; wean

jejunostomy feeds gradually.

- Teach small, frequent, protein-rich meals; upright positioning after eating; and separation of liquids from solids.
- Manage dumping syndrome stepwise: diet first, then viscosity agents/acarbose, then octreotide.
- Check B12, iron, folate, calcium, and vitamin D on a scheduled basis — deficiencies often emerge months to years after surgery; B12 replacement is lifelong after gastrectomy.
- Prescribe structured aerobic + resistance exercise (ideally starting at/before diagnosis) to counter sarcopenia.
- Refer for SLP-directed swallowing rehabilitation when dysphagia is present; keep a low threshold to rule out anastomotic stricture.
- Screen for psychosocial distress at every major transition and provide a formal survivorship care plan.

References

1. Weimann, A.; Braga, M.; Carli, F.; et al. ESPEN Practical Guideline: Clinical Nutrition in Surgery. *Clin. Nutr.* **2021**, 40, 4745–4761.
2. Kondrup, J.; Rasmussen, H.H.; Hamberg, O.; Stanga, Z. Nutritional Risk Screening (NRS 2002): A New Method Based on an Analysis of Controlled Clinical Trials. *Clin. Nutr.* **2003**, 22, 321–336.
3. Martin, L.; Birdsell, L.; Macdonald, N.; et al. Cancer Cachexia in the Age of Obesity: Skeletal Muscle Depletion Is a Powerful Prognostic Factor, Independent of Body Mass Index. *J. Clin. Oncol.* **2013**, 31, 1539–1547.
4. Scarpellini, E.; Arts, J.; Karamanolis, G.; et al. International Consensus on the Diagnosis and Management of Dumping Syndrome. *Nat. Rev. Endocrinol.* **2020**, 16, 448–466. https://doi.org/10.1038/s41574-020-0357-5..
5. Campbell, K.L.; Winters-Stone, K.M.; Wiskemann, J.; et al. Exercise Guidelines for Cancer Survivors: Consensus Statement from International Multidisciplinary Roundtable. *Med. Sci. Sports Exerc.* **2019**, 51, 2375–2390.
6. Ligibel, J.A.; Bloomquist, K.; Wolin, K.Y.; et al. Exercise, Diet, and Weight Management during Cancer Treatment: ASCO Guideline. *J. Clin. Oncol.* **2022**, 40. https://doi.org/10.1200/JCO.22.00687.
7. National Comprehensive Cancer Network. NCCN Clinical Practice Guidelines in Oncology: Distress Management, Version 1.2026.
8. Low, D.E.; Allum, W.; De Manzoni, G.; et al. Guidelines for Perioperative Care in Esophagectomy: Enhanced Recovery after Surgery (ERAS®) Society Recommendations. *World J. Surg.* **2019**, 43, 299–330.

References 1–8 correspond to references [28], [12], [7], [9], [1], [10], [2], and [27], respectively, in the main manuscript's reference list; they are renumbered here for standalone readability of this supplementary document.
